# Impact of containment measures on community mobility, daily confirmed cases, and mortality in the third wave of COVID-19 epidemic in Myanmar

**DOI:** 10.1186/s41182-022-00413-8

**Published:** 2022-03-11

**Authors:** Ye Minn Htun, Tun Tun Win, Nyan Htet Shan, Zin Thu Winn, Kaung Si Thu, Nyan Lin Maung, Pyae Phyo Aung, Htun Aung Kyaw, Hpone Pji Kyaw, Yan Naing Myint Soe, Myint Myat Ko, Zin Ko Aung, Kyaw Thiha Aung, Yan Paing Chit Lwin, Wai Yan, Phyo Tayza Soe, Kyaw Myo Tun

**Affiliations:** 1 Department of Prevention and Research Development of Hepatitis, AIDS and Other Viral Diseases, Health and Disease Control Unit, Nay Pyi Taw, 15011 Myanmar; 2 Department of Preventive and Social Medicine, Defence Services Medical Academy, Mingaladon, Yangon, Myanmar; 3 Outpatient Department, No. 1 Military Hospital (500 bedded), Meiktila, Mandalay, Myanmar; 4 Department of Research and Development, Defence Services Medical School, Hmawbi, Yangon, Myanmar

**Keywords:** Confirmed cases, Containment measures, COVID-19, Deaths, Mobility, Myanmar

## Abstract

In Myanmar, third wave of COVID-19 epidemic began with a surge of confirmed cases in the last week of May 2021. The laboratory-confirmed cases and deaths distinctly increased within 9 weeks. The government and the Ministry of Health adopted containment measures to flatten the peak of the epidemic and to suppress the disease transmission. The strictly containment measures: stay-at-home restrictions, school closure, and office closure have reduced the community mobility, confirmed cases and mortality. Therefore, the timely containment measures implemented by the government were important to reduce the transmission as observed in the third wave of COVID-19 epidemic in Myanmar.

**Supplementary Information:**

The online version contains supplementary material available at 10.1186/s41182-022-00413-8.

## Background

The current coronavirus disease 2019 (COVID*-*19) pandemic has led to a dramatic loss of human life and is still having a devastating impact on public health [1]. To prevent the further spread of the virus, many countries issued containment measures as community mitigation strategies including movement restrictions (e.g., border shutdown, travel restriction, stay-at-home, and lockdown), quarantine, and isolation.

### COVID-19 situation in Myanmar

In Myanmar, the first COVID-19 reported cases were identified on 23rd March 2020 and then the virus had rapidly spread to all states and regions. During the first wave of the COVID-19 epidemic, there were 379 confirmed cases, 359 recovered cases (94.7%), and 6 deaths (2.4%) [2]. After reported cases had abruptly increased in Rakhine State and Yangon Region on 19th August 2020, Myanmar was hit by the second wave of the COVID-19 epidemic [2, 3]. The majority of cases were transmitted locally and as a geographical distribution, Yangon Region was an epicenter of COVID-19 during the second wave. After performing the containment and mitigation measures (such as stay-at-home, lockdown, ban entry for all countries) by the government to prevent the disease spread, the new confirmed cases were reduced consequently. Ministry of Health (MOH) reported 143,318 confirmed cases with 132,264 recovered cases (92.2%) and 3216 deaths (2.2%) through 26th May 2021 [2].

Since 27th May 2021, the confirmed cases gradually increased again and the two reported COVID-19 clusters were identified in Bago Region (Letpadan cluster: 126 confirmed cases) and Yangon Region (Hlegu cluster: 246 confirmed cases) during the second week of July 2021. These clusters of infections played a critical role in modifying patterns of COVID-19 transmission, which exponentially increases the number of cases. Subsequently, Bago and Yangon were the regions with the highest detected cases in the early stage of the third wave. On 15th June 2021, MOH reported that Alpha: B.1.1.7 and Delta: B.1.617.2 (Variants of Concern) and Kappa: B.1.617.1 (former Variants of Interest) were detected in 11 confirmed cases as stated by the Defence Services Medical Research Centre. On 22nd July 2021, MOH also announced that Beta: B.1.351 variant (Variants of Concern) was newly detected in Myanmar. As the COVID-19 disease surveillance, on 31st August 2021, MOH stated that Delta variants were detected again in 15 confirmed cases (five cases in Nay Pyi Taw, two cases in Yangon, two cases in Mandalay, each case in Sittwe, Taungoo, Lashio, Mawlamyine, Monywa, and Kalay).

The emerging variants result in increased transmissibility and more serious illness (such as increased hospitalizations or deaths). In addition, they also affect diagnostic identification failures, which can potentially delay the diagnosis and treatment, exhibit decreased susceptibility to treatment including antivirals, monoclonal antibodies, and convalescent plasma, possess the ability to cause reinfection in previously infected individuals, and vaccine breakthrough cases in fully vaccinated individuals [4]. Hence, the community spread in the third wave was faster than the second wave in Myanmar. As of 24th November 2021, MOH reported that the total confirmed cases were 519,731 cases with 493,399 recoveries (94.9%) and 19,049 deaths (3.7%) [2]. In this communication, we describe the impact of containment measures on community mobility, daily confirmed cases, and mortality in the third wave of COVID-19 epidemic in Myanmar.

### Data retrieval and analysis

Daily COVID-19 data (from 27th May to 24th November 2021) were collated and retrieved from the daily reports of COVID-19 surveillance that can be accessed at Coronavirus Disease 2019 (COVID-19) Surveillance Dashboard (Myanmar) [2]. Daily confirmed cases, deaths, specimens tested were expressed as epidemic curves with a 7-day moving average and daily test positivity. The degree of change in confirmed cases and deaths over time (weekly) was expressed as percent changes.

Google COVID-19 Community Mobility Reports have provided a data set regarding mobility trends, which measures changes in population movements during the COVID-19 outbreak in each country. The six mobility patterns of population, across different categories of places, such as retail and recreation, groceries and pharmacies, parks, transit stations, workplaces, and residential, were extracted to examine the nationwide and local lockdown decisions and social-distancing requirements to reduce the transmission of COVID-19. This regularly updated data set of population movements that have changed throughout the pandemic was provided by apps, such as Google Maps [5]. Pearson correlation coefficient (*r*) was used to measure the strength of relationships between percent changes of mobility and daily confirmed cases. IBM SPSS Statistics for Windows, Version 23.0 (Armonk, NY: IBM Corp) was used in analyzing the data and *p* value was set at 0.05 for the level of significance.

### Third wave of COVID-19 epidemic in Myanmar

**Fig. 1:**
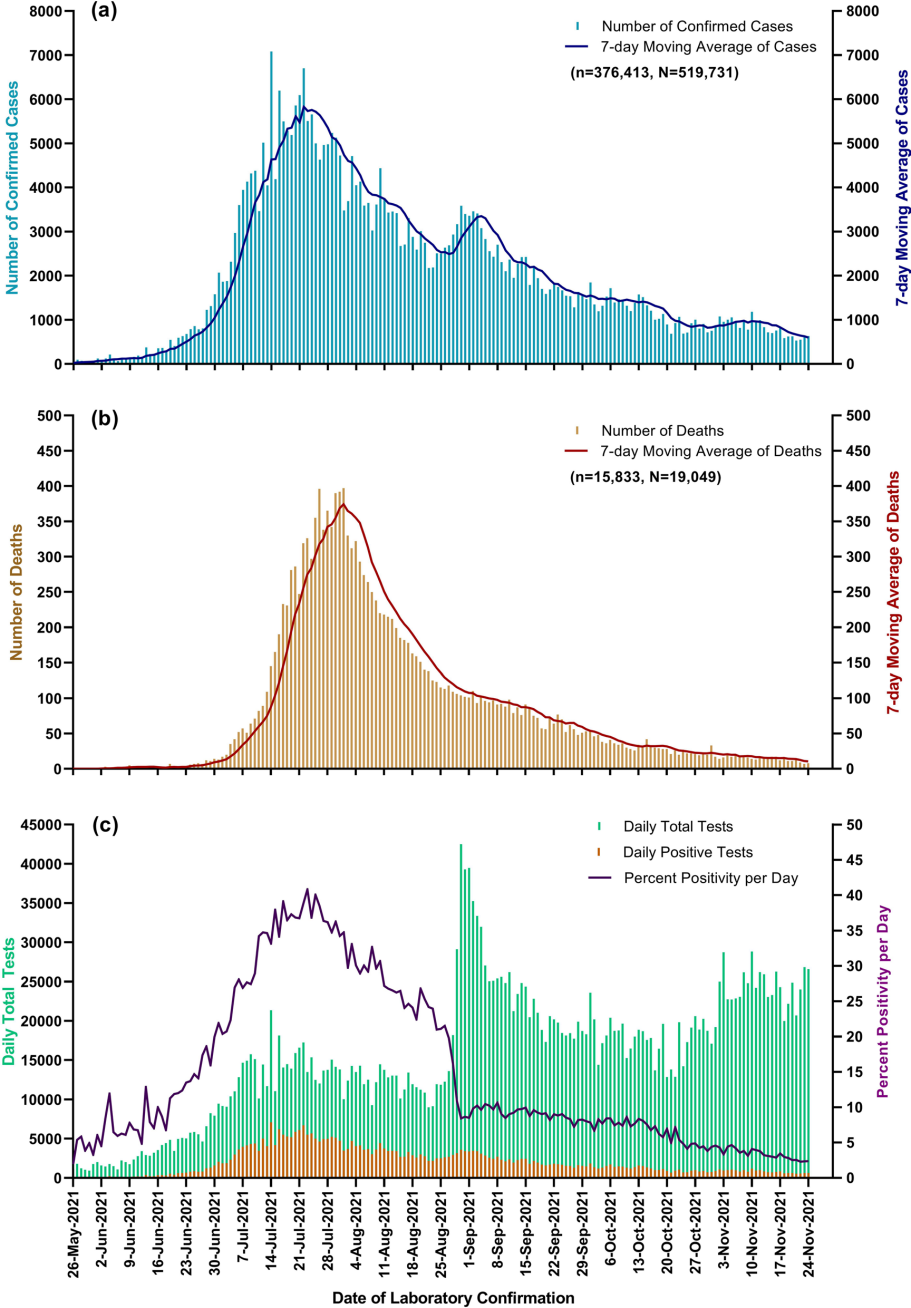
Epidemic curves of the third wave of epidemic in Myanmar, starting from 27th May to 24th November 2021 (**a**) Epidemic curves of daily and 7-day moving average of confirmed cases, *N*: total confirmed cases and *n*: number of confirmed cases from 27th May 2021 (**b**) Epidemic curves of daily and 7-day moving average of deaths, N: total deaths and *n*: number of deaths from 27th May 2021 (**c**) Epidemic curves of daily total tests, daily positive tests and percent positivity per day

In the third wave of the epidemic, MOH reported that more than 2000 daily confirmed cases were detected starting from 1st July 2021, more than 4000 daily confirmed cases starting from 8th July 2021, and more than 6000 daily confirmed cases starting from 14th July 2021 (Fig. 1a). For the COVID-19 related deaths, more than 100 daily deaths were reported starting from 13th July 2021, more than 200 daily deaths starting from 17th July 2021, and more than 300 deaths starting from 22nd July 2021 (Fig. 1b). The third wave of the COVID-19 epidemic took a peak in the fourth week of July (on 22nd July) 2021 and the length of the critical period (from the start of the wave to its peak) was 56 days. It was longer than South Korea and Italy (44 days); however, shorter than Sweden (90 days), India, and Nepal (90–130 days), by the findings of the study done from January to May 2020 [6].

The suspected persons with acute onset of sign and symptoms and those who were with fever, symptoms of severe acute respiratory disease within last 10 days, the persons with travelling history within last 14 days, and the persons with a history of close contact within the past 14 days were reported to the respective State and Regional or District or Township Health Department. Then, the specimens were tested at designated laboratories. At the start of the third wave, the daily total test was below 2000 and the percent positivity of the test was around 5.0%. To detect more COVID-19 positive patients in the community, to reduce the secondary cases, and to get more accessibility of diagnostic testing, MOH expanded the testing capacity at both public and private health sectors. Hence, MOH could perform more than 2000 daily total tests starting from 7th June 2021, more than 10,000 daily total tests starting from 4th July 2021, and more than 20,000 daily total tests starting from 30th August 2021. In the last week of July, the percent positivity was more than 30% with a range of 34.2–40.8%. Then, it was reduced continuously and remained at less than 5% starting from 24th October 2021 (Fig. 1c).

### Containment measures in the third wave

To contain the spread of COVID-19, it is vital to understand the circumstances of the SARS-CoV-2 transmission and the preventive measures available to reduce the spread of the disease. MOH performed the mask campaign and health education, quarantine and screening the people who came back from foreign countries, development of community test centers, expansion of testing and treatment facilities, providing medical care for the confirmed cases at designated hospitals and treatment centers depending on disease severity, installation of oxygen concentrators, extension of school and office closures in high-risk areas, extension of the precautionary restriction measures relating to control of the COVID-19 pandemic for all travelers visiting Myanmar, and temporary suspension of all types of visa and visa exemption. To reduce the risk of disease spread effectively, Myanmar is making an effort to improve the vaccination coverage according to National Deployment Plan, and vaccination program was implemented with the priority target groups (such as the people over the age of 65, 55, and 45 years, health workers, government staff, the students with the age of older than 12 years, ethnic minority and the migrants).

**Fig. 2:**
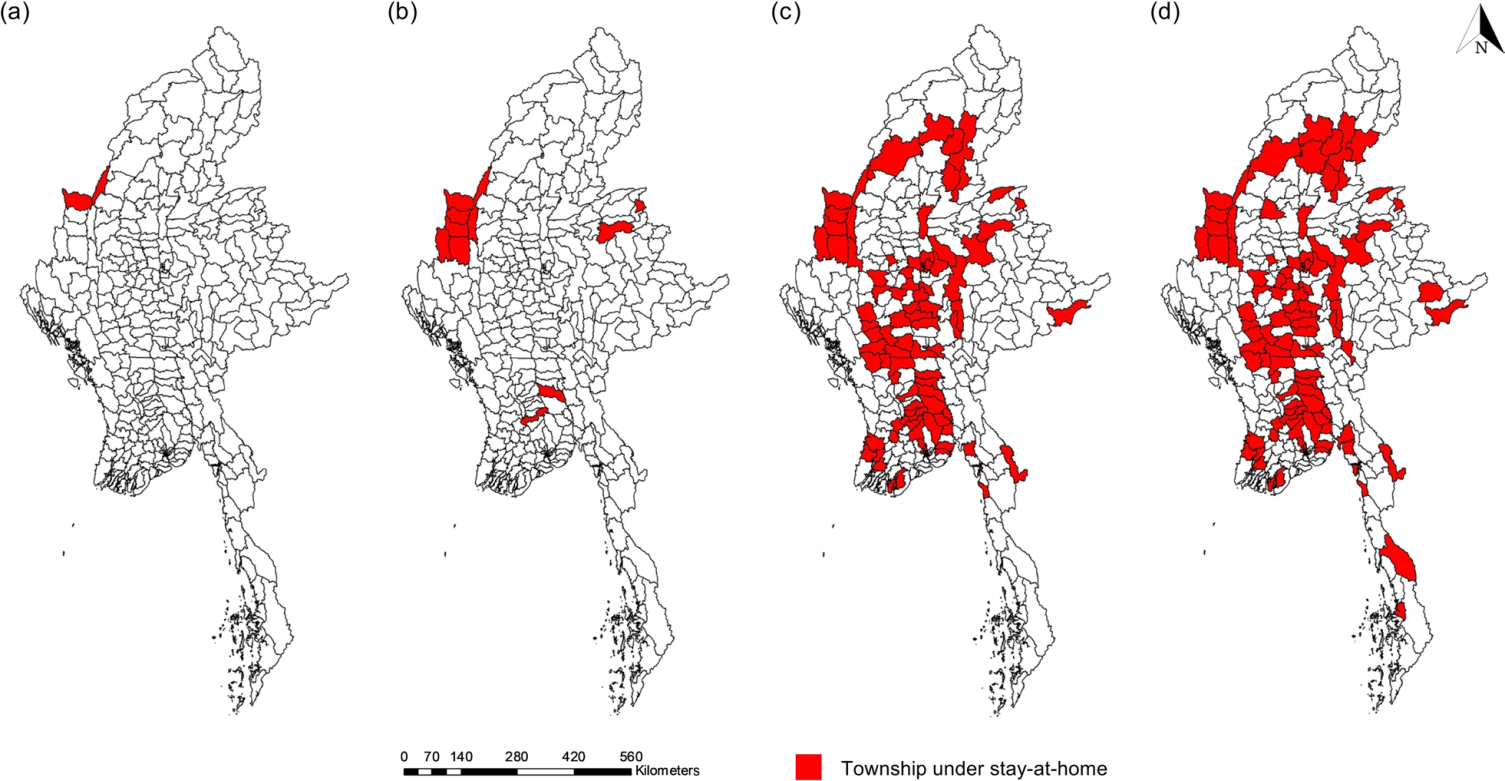
Townships under stay-at-home in the third wave of COVID-19 epidemic in Myanmar from 29th May to 25th August 2021 (**a**) two townships under stay-at-home in May 2021 (**b**) 11 townships under stay-at-home from May to June 2021, **c** 108 townships under stay-at-home from May to July 2021, **d** 119 townships under stay-at-home from May to August 2021

For the response to the COVID-19 epidemic, the Myanmar government enacted a range of COVID-19 containment measures. MOH imposed a stay-at-home restriction in the highest case detected areas and there were 119 stay-at-home townships from 14 states and regions starting from 29th May to 25th August 2021 (Fig. 2). In addition, the government adopted additional containment measures such as school closure (started from 12th July to 31st October 2021) and office closure (started from 17th July to 10th September 2021) (see Additional file 1 for the containment measures in the third wave by the start date).

### Containment measures and community mobility

**Fig. 3:**
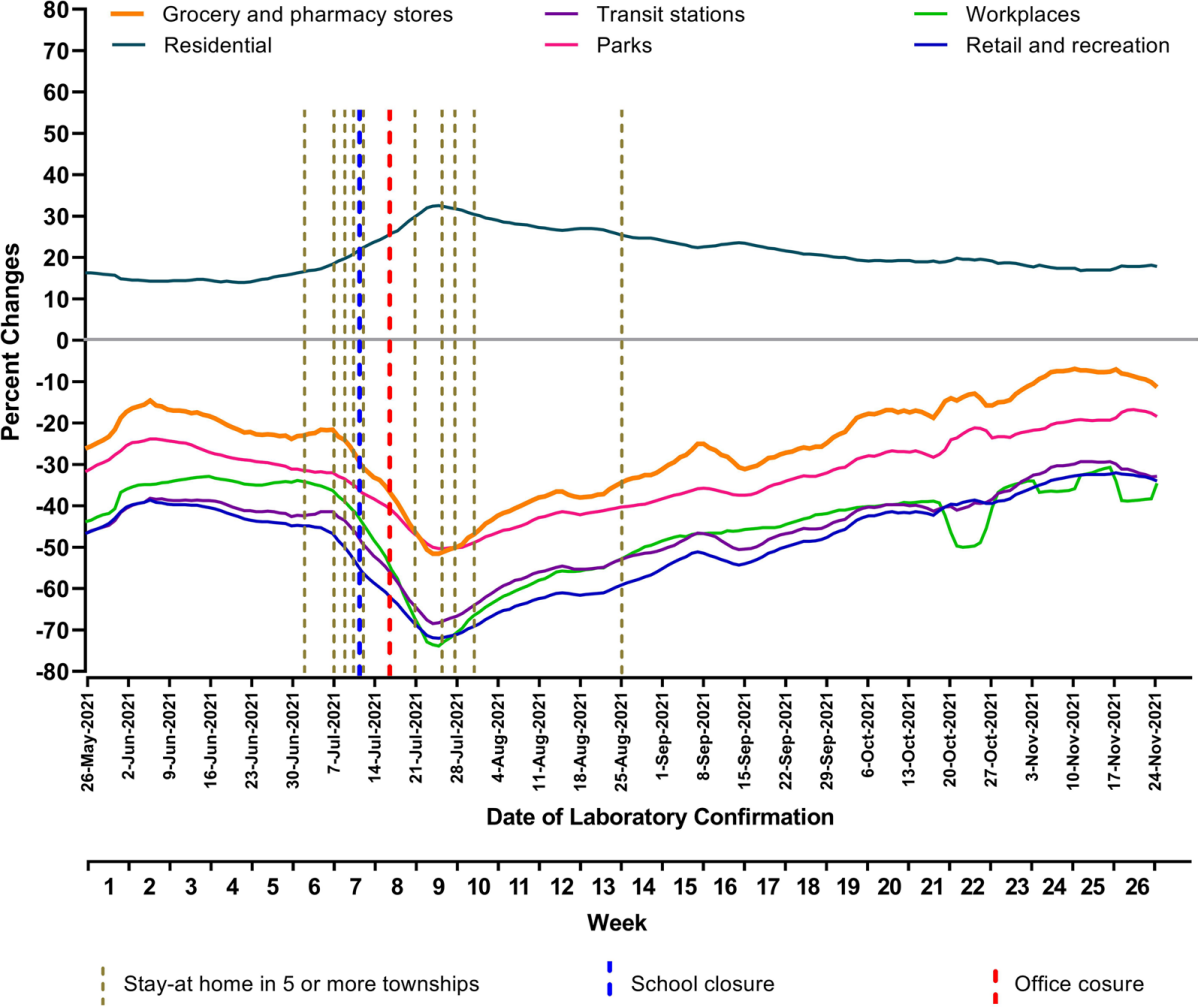
Containment measures and community mobility trends in the third wave of the COVID-19 epidemic in Myanmar. Community mobility changes in the six categories were shown compared to baseline days of the median value for the 5-week period from January 3 to February 6 2020. The indexes were smoothed to the rolling 7-day average. Data source: https://ourworldindata.org/coronavirus

In the third wave of COVID-19 epidemic, the population movement around residential area, which was changed in the duration of time spent at home, increased during a period of most containment measures (weeks 7–9) (Fig. 3). Going to workplace activity, the movement of people around the retail shop, parks, transit stations, and grocery and pharmacy stores decreased starting from week 7 to week 9.

These changes could be explained by the fact that high enforcement of the restrictions by the government and concern about the severity of diseases might reduce the mobility of people. The factors such as people’s will and ability to observe these restrictions, level of education, and ability to maintain basic consumption expenditure without working affected the extent of the restrictions. In addition, it could be argued that the stricter restrictions observed, the higher reduction in people’s mobility in public areas and an increase in time spent in residential areas [7]. However, there is a variation between countries in the reduction of the percent changes among visitors due to different containment measures and policies.

### Containment measures, detection of daily confirmed cases and mortality

**Fig. 4:**
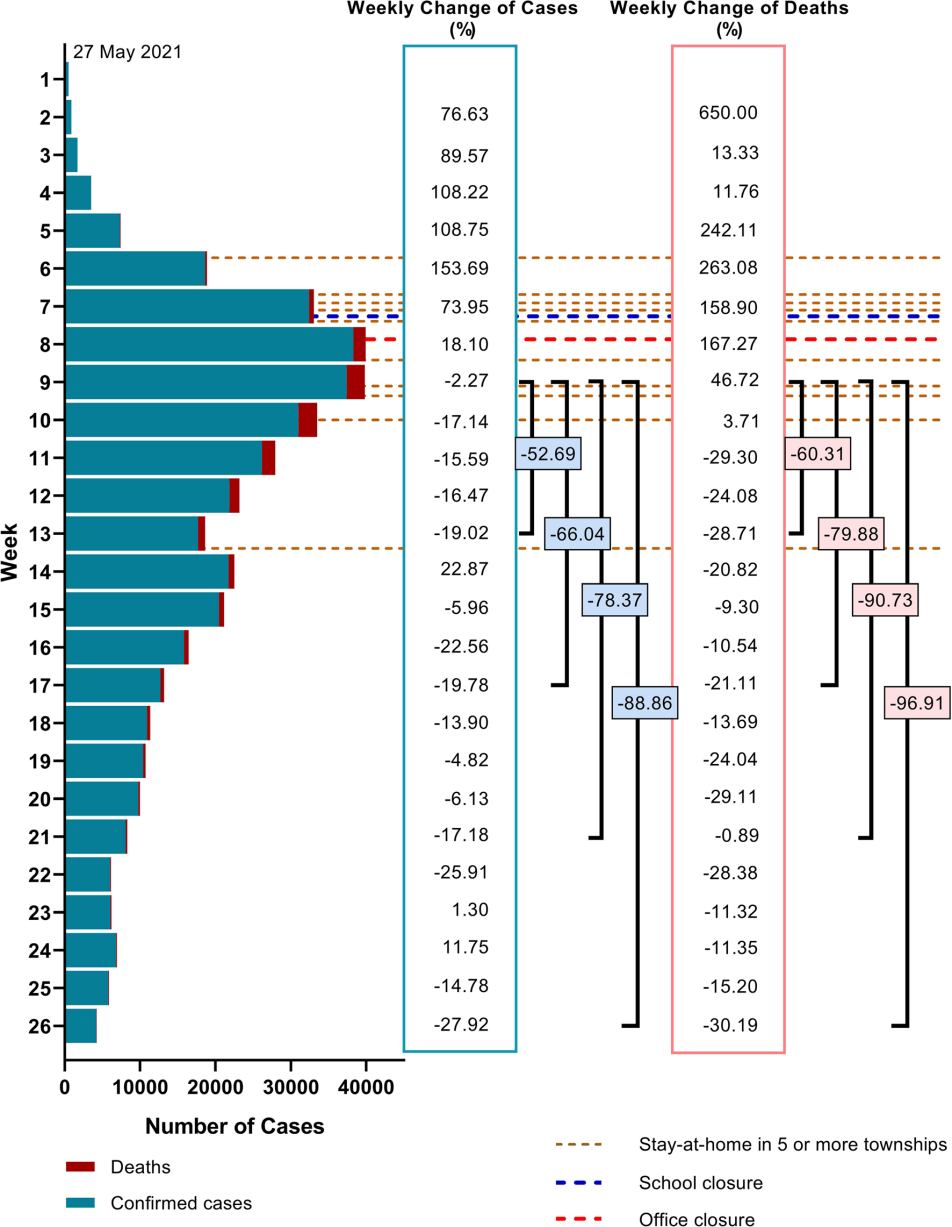
Containment measures, changes of confirmed cases and deaths in the third wave of COVID-19 epidemic in Myanmar

In the third wave of COVID-19 in Myanmar, the weekly percent changes of confirmed cases and deaths were positive values from week 1–8 and week 1–10, respectively (Fig. 4). After a period of most containment measures (weeks 7–9), the weekly percent changes of confirmed cases and deaths were negative values. Confirmed cases reduced 52.69% at 4 weeks, 66.04% at 8 weeks, 78.37% at 12 weeks, and 88.86% at 17 weeks, from the peak of the epidemic (week 9). COVID-19 related deaths also reduced 60.31% at 4 weeks, 79.88% at 8 weeks, 90.73% at 12 weeks, and 96.91% at 17 weeks, from the peak of the epidemic.

It could be suggested that the containment measures had a positive impact on COVID-19 infection and deaths. An ecological study stated that a significant reduction in daily confirmed cases was documented in South Africa, Germany, Spain, Italy, and New Zealand with lockdown implementation [8]. Another previous study also approved that the early introduction of combined public health and social measures was effective with no local transmissions [9]. However, it could be mindful that the responses to movement restrictions depend on the socioeconomic conditions of the population.

There were statistical significant relationships between daily confirmed cases and six categories of community mobility (residential *r* = 0.84, *p* < 0.001: grocery and pharmacy stores *r* = − 0.82, *p* < 0.001: parks *r* = − 0.84, *p* < 0.001: workplaces *r* = − 0.79, *p* < 0.001: transit stations *r* = − 0.82, *p* < 0.001, and retail and recreation *r* = − 0.86, *p* < 0.001) (see Additional file 2 for the figure of relationship between daily confirmed cases and percent change of community mobility). It was in line with the findings of a study assessing the effects of human mobility restriction on COVID-19 prevalence in the Global South countries which were less economically developed countries in the regions of Latin America, Asia, Africa, and Oceania, stated that there was a relationship between percent change of visitors and total cases of COVID-19 [10].

## Conclusions

This finding provides the broadest observational assessment on timely containment measures in the reduction of COVID-19 transmission. It can also be suggested that early adoption of NPIs with strict restrictions might impact the population movement, daily confirmed cases, and death. Moreover, the disease surveillance should continue to prevent further spread, even though the rapid return to normal mobility after the release of the containment measures. The tool provided by Google may be helpful to understand population response to various control measures, but more research is needed to understand the social, economic, and cultural concerns that are responsible for the differences in adherence to containment measures.

## Supplementary Information

**Additional file 1: Table S1.** Containment measures in the third wave of COVID-19 epidemic in Myanmar, by the start date.

**Additional file 2: Figure S1.** The relationship between daily confirmed cases and percent change of community mobility in the third wave of COVID-19 epidemic in Myanmar.

## Data Availability

All data used are publicly available, and sources are cited throughout.

## Abbreviations

COVID*-*19: Coronavirus Disease 2019
MOH: Ministry of Health
NPIs: Nonpharmaceutical Interventions
SARS-CoV-2: Severe Acute Respiratory Syndrome Coronavirus 2
