# Supplementary material for: Impact of containment measures on community mobility, daily confirmed cases, and mortality in the third wave of COVID-19 epidemic in Myanmar
Source: Trop Med Health. 2022 Mar 11;50:23. doi: 10.1186/s41182-022-00413-8 (PMC8913326; doi:10.1186/s41182-022-00413-8)
Supplement: Supplementary file 1 — Additional file 1: Table S1. Containment measures in the third wave of COVID-19 epidemic in Myanmar, by the start date. [file 41182_2022_413_MOESM1_ESM.docx]

**Table S1.** Containment measures in the third wave of COVID-19 epidemic in Myanmar, by the start date

|  | Start date | Number of Township | State and Region |
| --- | --- | --- | --- |
| Stay-at-home | |  | |
|  | 29 May 2021 | 2 | Chin, Sagaing |
|  | 3 Jun 2021 | 1 | Sagaing |
|  | 5 Jun 2021 | 4 | Chin |
|  | 19 Jun 2021 | 1 | Bago |
|  | 22 Jun 2021 | 1 | Bago |
|  | 27 Jun 2021 | 1 | Shan |
|  | 28 Jun 2021 | 1 | Shan |
|  | 2 July 2021 | 9 | Bago, Mandalay |
|  | 3 July 2021 | 3 | Mandalay, Sagaing, Shan |
|  | 4 July 2021 | 3 | Ayeyarwady |
|  | 7 July 2021 | 5 | Bago, Nay Pyi Taw |
|  | 8 July 2021 | 14 | Magway, Mon, Yangon |
|  | 12 July 2021 | 18 | Bago, Yangon |
|  | 13 July 2021 | 11 | Ayeyarwady, Kachin, Kayin, Magway, Sagaing, Shan |
|  | 21 July 2021 | 12 | Kachin, Magway |
|  | 26 July 2021 | 7 | Magway, Nay Pyi Taw, Shan |
|  | 28 July 2021 | 5 | Ayeyarwady, Bago, Mandalay |
|  | 31 July 2021 | 10 | Bago, Mandalay |
|  | 4 August 2021 | 2 | Mandalay, Mon |
|  | 20 August 2021 | 1 | Shan |
|  | 23 August 2021 | 3 | Nay Pyi Taw, Tanintharyi |
|  | 25 August 2021 | 5 | Kachin, Kayah, Mon, Sagaing |
| School closure | | | |
|  | 12 July 2021 | The whole Union | |
| Office closure | | | |
|  | 17 July 2021 | The whole Union | |
