# Supplementary material for: Impact of containment measures on community mobility, daily confirmed cases, and mortality in the third wave of COVID-19 epidemic in Myanmar
Source: Trop Med Health. 2022 Mar 11;50:23. doi: 10.1186/s41182-022-00413-8 (PMC8913326; doi:10.1186/s41182-022-00413-8)
Supplement: Supplementary file 2 — Additional file 2: Figure S1. The relationship between daily confirmed cases and percent change of community mobility in the third wave of COVID-19 epidemic in Myanmar. [file 41182_2022_413_MOESM2_ESM.docx]

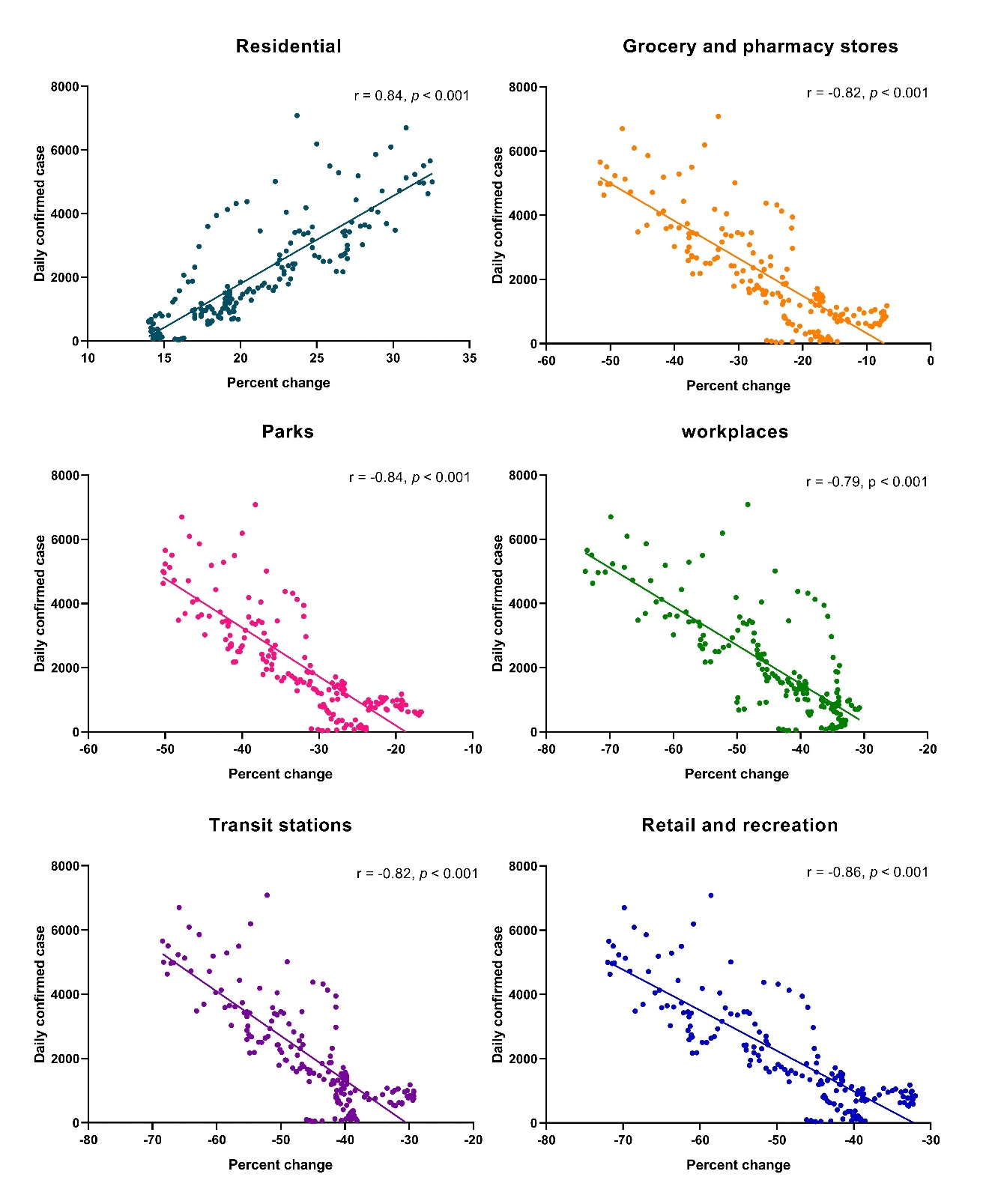


**Fig S1.** The relationship between daily confirmed cases and percent change of community mobility in the third wave of COVID-19 epidemic in Myanmar
